# Tumor-derived exosomal linc00881 induces lung fibroblast activation and promotes osteosarcoma lung migration

**DOI:** 10.1186/s12935-023-03121-3

**Published:** 2023-11-21

**Authors:** Xinyu Chang, Qiuyu Tan, Jinwen Xu, Xu Wu, Ying Wang, Yuan Zhang, Hao Zhang, Haijun Liu, Liang Yan

**Affiliations:** 1 Department of Oncology, The First Affiliated Hospital of Wannan Medical College (Yijishan Hospital of Wannan Medical College), Wuhu, 241002 Anhui China; 2 Provincial Key Laboratory of Biological Macro-Molecules Research, WanNan Medical College, Wuhu, 241002 Anhui China; 3 Department of Orthopedics Trauma, The First Affiliated Hospital of Naval Medical University, Shanghai, 200433 China

**Keywords:** linc00881, MMP2, Lung fibroblast activation, Lung migration, Osteosarcoma

## Abstract

Osteosarcoma (OS) commonly metastasizes to the lung, yet the underlying molecular mechanisms remain poorly understood. Exosomes play a crucial role in tumor migration, including OS lung migration. However, the underlying mechanism by which exosome-derived long non-coding RNAs (lncRNAs) contribute to lung migration in osteosarcoma (OS) remains unclear. This study presents a newly discovered lncRNA, linc00881, derived from OS exosomes. Our study shows that linc00881 promotes the migration of OS cells to the lung and induces the conversion of normal lung fibroblasts into cancer-associated fibroblasts (CAFs). Subsequently, we found that exosomal linc00881 secreted by OS cells can regulate the expression of matrix metalloproteinase 2 (MMP2) in HFL-1 cells by sponging miR-29c-3p, thereby activating the NF-κB signaling in lung fibroblasts. Finally, we discovered that pro-inflammatory cytokines, namely IL-1β, IL-6, and IL-8, were secreted through the linc00881/miR-29c-3p/MMP2 axis. These results suggest that OS-derived exosomes can mediate the intercellular crosstalk between OS cells and lung fibroblasts, ultimately impacting OS lung migration. Our study provides a potential target for the treatment of OS lung migration.

**Graphical Abstract:**

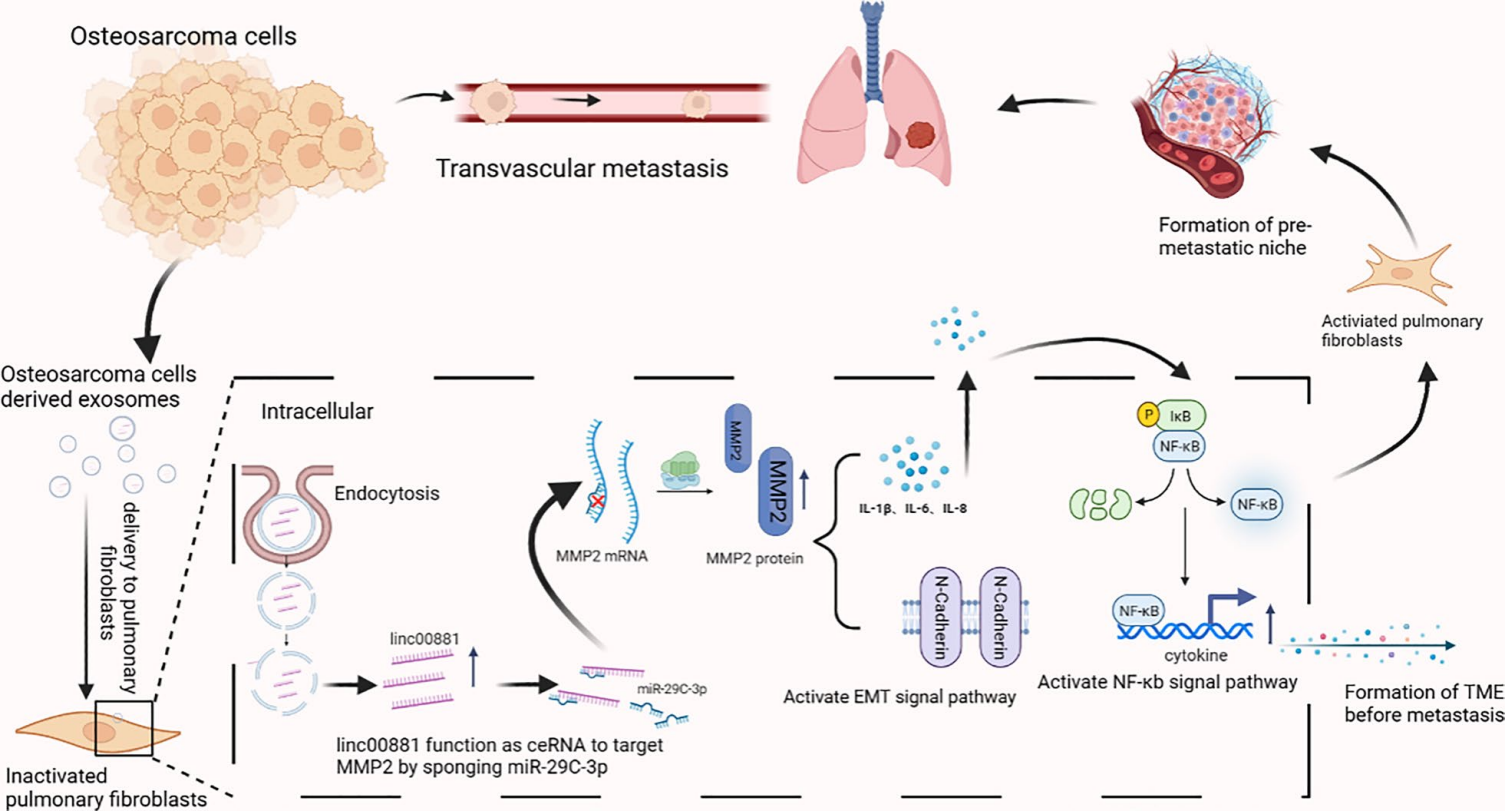

**Supplementary Information:**

The online version contains supplementary material available at 10.1186/s12935-023-03121-3.

## Introduction

Osteosarcoma (OS) is the most prevalent form of primary bone cancer, and it frequently metastasizes to the lungs and other distant bones [1]. Secondary lung cancer is the primary cause of death among patients with OS [2]. Surgery and systemic chemotherapy are the main treatment options for OS; however, the effectiveness remains unsatisfactory. According to the statistics, the 5-year survival rate, which was examined utilizing a multidisciplinary approach, ranges from 60 to 70% [3]. Currently, the factors and pathways that regulate the process of OS migration remain unclear.

Exosomes are extracellular vesicles ranging in size from 30 to 150 nm and can carry various types of nucleic acids, such as messenger RNA (mRNA), micro RNA (miRNA), transfer RNA (tRNA), and long non-coding RNA (lncRNA) [4]. Exosomes facilitate intercellular communication by transferring RNA molecules [5]. Recent research has shown that exosomal lncRNA plays a significant role in tumor metastasis, and the most common mechanism is the competing endogenous RNAs (ceRNA) mechanism [6, 7]. For example, tumor-derived exosomal lncRNA SOX2OT promotes the bone metastasis of non-small cell lung cancer by adsorbing miRNA miR-194-5p [8]. Macrophage-derived exosomal lncRNA LIFR-AS1 in OS promotes OS progression by adsorbing miR-29a [9]. Studies have shown that OS-derived exosomes are abundant in lncRNAs. Furthermore, it has been demonstrated that these exosomal lncRNAs play significant roles in the spread and resistance to chemotherapy in different types of cancer [10–12]. Further investigation is imperative to elucidate the involvement of exosomal lncRNAs in the metastasis of OS to the lungs.

Therapeutic strategies targeting components in the tumor microenvironment are effective options against tumor metastasis [13]. Previous studies suggest that CAFs, which are a subtype of activated fibroblasts, have a significant impact on promoting tumor metastasis [14]. CAFs exhibit high heterogeneity as they originate from different sources, and α-smooth muscle actin (α-SMA) is widely employed to identify the activation of CAFs [15]. Furthermore, CAFs upregulate the expression of pro-inflammatory genes such as IL-1β, IL-6, and IL-8, thereby playing a crucial role in modulating the inflammatory microenvironment [16]. However, there is a lack of studies exploring the role of CAFs in osteosarcoma lung migration.

In this study, we found that OS-derived exosomes promoted the migration of OS cells to the lung fibroblasts. Additionally, we observed an elevation in the expression of α-SMA in lung fibroblasts. Subsequently, we found that OS exosome-derived linc00881 may play an important part in the lung migration of OS through high throughput sequencing. Subsequently, we found that linc00881 was highly expressed in lung fibroblasts upon stimulation with exosomes released by two types of OS. Finally, our study demonstrated that OS exosome-derived linc00881, which interferes with the extracellular matrix of lung fibroblasts and induces their transformation into CAFs by modulating the miR-29c-3p/MMP2 axis in lung fibroblasts, thereby enhancing the migration of OS cells to the lung. This study highlights the significant role of linc00881 in OS lung migration and establishes a novel theoretical basis for investigating its involvement in the treatment of OS lung metastasis.

## Materials and methods

### Cell culture and transfection

Cell lines derived from humans (MG-63, HOS) were obtained from Fuheng Biotechnology Co., Ltd (Shanghai, China). Both types of OS cells were grown in DMEM (Gibco, CA, USA) medium with the addition of 10% exosome-depleted fetal bovine serum (exoFBS) (Systembio, CA, USA) at 37 ℃ and 5% CO_2_. 2 × 10^5^ OS cells were placed on 6-well plates. Upon reaching 50–60% confluency, the corresponding RNA particles were transfected using INTERFERin (Polyplus-transfection, Alsace, France), and DNA plasmids were transfected using MIR2300 TransIT^®^-LT1 Transfection Reagent (Mirus Bio, WI, USA) following the manufacturers' instructions. The linc00881 over-expression plasmid and siRNA were acquired from Genepharma (Shanghai, China). Mimics and inhibitors of MiR-29c-3p were acquired from RiboBio (Guangzhou, China).

### Isolation of exosomes

Exosomes were isolated using the exosome extraction kit (Systembio, CA, USA) in accordance with the guidelines provided by the manufacturer. A total of 15 mL cell culture was collected and subsequently centrifuged at 3000 rpm for 15 min. The liquid above was moved to a fresh tube for centrifugation, and 3 mL of exosome extraction reagent was added. After mixing, the mixture was vertically placed at 4 ℃ overnight. Afterward, the mixture was centrifuged at 1500 rpm for 30 min. The supernatant was discarded, and the pellet was suspended using 300 μL of 1 × PBS (Phosphate Buffered Saline, PBS).

### Transmission electron microscopy

5–10 µL of the resuspended exosome solution (previously fixed with 2.5% glutaraldehyde) were applied onto the copper grid and adsorbed at room temperature for approximately 5 min. The excessive liquid was carefully removed by blotting with filter paper. Afterward, 10 μL of staining solutions (saturated uranyl acetate) were added to the copper grid and stained for 1 min at room temperature. Excess dye was absorbed with filter paper. A drop of ddH_2_O was added to the copper grid and kept at room temperature for 5 min. The copper mesh was air-dried after a second wash. Micrographs were captured using a transmission electron microscope JEM-1230 (JEOL, TKY, Japan) operating at 80 kV.

### Nanoparticle tracking analysis

Nanoparticle tracking analysis was conducted as previously described [17]. We used the Nanosight NS300 (Malvern Instruments, Malvern, UK) to measure the exosomes’ concentration and size distribution. Data were assessed using NTA analytical software (version 2.3).

### Identification and analysis of lncRNAs

Transcript quantification was carried out using Stringtie (version 1.3.0) [18, 19]. New transcripts were acquired by comparing the assembled transcripts with known human protein-coding transcripts using gfcompare (version: 0.9.8). Presumed lncRNAs were identified as new transcripts that met the following criteria: length ≥ 200 bp; number of exons ≥ 2; ORF ≤ 300 bp; lacking or having limited protein-coding potential (CPC score < 0 [20] & CNCI score < 0 [21] & no significant resemblance to Pfam database [22]). Finally, to acquire a unique collection of lncRNAs, we employed gffcompare to combine the lincRNAs discovered from RNA-seq data with the previously recorded lncRNAs in the NONCODE v5 database.

### qRT-PCR

The RNAfast200 kit (Fastagene, Shanghai, China) was used to extract total cellular RNA according to the instructions provided by the manufacturer. SuperScript IV Reverse Transcriptase (Thermo, MA, USA) was used to reverse RNA transcription into cDNA. Subsequently, SYBR™ Select Master Mix (Thermo, MA, USA) was employed to detect the expression of the target gene using cDNA as the template. Table 1 displays the primer sequences.

**Table 1:** Sequences of primers

Primer	Forward	Reverse
MMP2	5ʹ-TGATGGCATCGCTCAGATCC-3ʹ	5ʹ-GGCCTCGTATACCGCATCAA-3ʹ
GAPDH	5ʹ-CGAGCCACATCGCTCAGACA-3ʹ	5ʹ-GTGGTGAAGACGCCAGTGGA-3ʹ
IL-1β	5ʹ-AGCCATGGCAGAAGTACCTG-3ʹ	5ʹ-TGTCCATGGCCACACAACT-3ʹ
IL-6	5ʹ-TTCGGTCCAGTTGCCTTCTC-3ʹ	5ʹ-TGTTTTCTGCCAGTGCCTCT-3ʹ
IL-8	5ʹ-TTCTGCAGCTCTGTGTGAAG-3ʹ	5ʹ-TCTCAGCCCTCTTCAAAAACTT-3ʹ
α-SAM	5ʹ-CAGCCAAGCACTGTCAGGA-3ʹ	5ʹ-GTCACCCACGTAGCTGTCTT-3ʹ
Linc00881	5ʹ-AGGTGCTGTATTGGCTCTTGAC-3ʹ	5ʹ-TCACTCACAGCAGATGAATCCC-3ʹ

### RNA pull-down assay

RiboBio (located in Guangzhou, China) synthesized the biotin-labeled miR-29c-3p and control probe, transfected into the 293T cells. After transfection, cells were lysed with NP-40 Lysis Buffer (Beyotime, Beijing, China) according to the instructions provided by the manufacturer after 24 h. Subsequently, an RNA pull-down assay was performed as previously described [23].

### Fluorescence in situ hybridization (FISH) assay

The FISH assay was employed to determine the localization of linc00881 in HFL-1 cells. The linc00881 targeting probe was synthesized by RiboBio (Guangzhou, China). Slides were used to seed HFL-1 cells, which were then incubated for 24 h. Cells were rinsed 2 times using (pH 7.2–7.4). Cells were pre-treated with a prehybridization buffer after a fixed infiltration. Next, the culture medium containing the linc00881 probe was added for hybridization in the dark for 12 h. Cells were then flushed with the 4 × , 2 × , and 1 × sodium citrate saline buffer at 42 ℃. Subsequently, the cells were stained using DAPI (4', 6-diamidino-2-phenylindole, DAPI) and captured using a Zeiss laser scanning confocal microscope (Oberkochen, Germany).

### Western blot and antibody

Protein extraction from cells or exosomes was performed using RIPA (Beyotime, Beijing, China). After separating using an SDS-PAGE gel, the proteins were transferred onto a PVDF membrane (Roche, BSKL, CH). The membranes were first blocked with 5% BSA (bovine serum albumin) for 1 h and then incubated at 4 °C overnight with primary antibodies, respectively, including N Cadherin (1:1000 dilution) (#4061, CST, MA, USA), Exosomal Marker Antibody Sampler Kit (1:1000 dilution) (#74220, CST, MA, USA), GAPDH (1:1500 dilution) (E0520, Santa, CA, USA), Phospho-NF-kappaB(1:1000 dilution) (#3033 T, CST, MA, USA), IκBα(1:500 dilution) (#4812S, CST, MA, USA), NF-kapoaB (1:1000 dilution) (#8242 T, CST, MA, USA), and MMP2 (1:500 dilution) (#40994S, CST, MA, USA). Anti-IL-6 neutralizing antibodies were purchased from proteintech (Wuhan, China). Subsequently, the membranes were exposed to a secondary antibody at ambient temperature for 1 h. The blots were then visualized using the SuperSignal West Atto (A38555, Thermo, MA, USA).

### Transwell assay

Cell migration ability was assessed through transwell analysis utilizing transwell chambers with 24 wells and polycarbonate membranes measuring 8 μm (Millipore, MA, USA)). HFL-1 cells were seeded on the 24-well chamber filled with 500 μL of F12K medium supplemented with 10% FBS. The lower chamber was then inserted in 24 well chambers, and 2 × 10^4^ OS cells were added to the chamber with 200 μL of serum-free F12K medium. After being incubated at 37 ℃ for 24 h, the cells on the upper membrane’s surface were scraped away. The migrated cells on the membrane's surface were then fixed using 4% methanol and stained with 0.1% crystal violet (Beyotime, Beijing, China). Micrographs were captured using the inverted microscope (Nikon, Japan), and the count of migrated cells was performed in a minimum of three random microscopic fields/well (100 ×).

### Animal studies

To explore the function of exosomes in a lung migration model, nude mice (4 weeks old) were acquired from Gempharmatech (Nanjing, China). Then, 2 × 10^7^ of GFP (Green fluorescent protein)-labeled MG63 cells were injected intravenously into the nude mice through the tail vein. The mice were then divided into two groups randomly. Subsequently, the test group received intravenous injections of 200 μL of exosomes every other day, while the control group received 200 mL 1 × PBS injections. One month later, ex vivo bioluminescent imaging was performed to measure lung metastasis using IVIS Lumina series III (PerkinElmer, MA, USA). The Animal Use and Care Committee of Wannan Medical College approved this animal experiment (No. LLSC-2022-224).

### Exosomes tracing

The two OS cell lines used in this study were pretreated with Dil (C1991S, Beyotime, Beijing, China) to track the exosomes. Later, the pre-treated exosomes were incubated with HFL-1 cells which were pre-treated with DAPI (C1002, Beyotime, Beijing, China), followed by visualization by a confocal laser scanning microscope (CLSM) (TCS-SP8, Leica, IL, USA).

### Statistical analysis

All experiments were utilized in triplicates or more, and representative images were selected for visualization in this paper. A one-way ANOVA was utilized to determine the differences between groups, while a t-test was applied to establish the disparity between two particular groups (e.g., T-test) (*p* < 0.05).

## Results

### OS-derived exosomes induce lung fibroblasts activation and promote OS lung migration

**Fig. 1:**
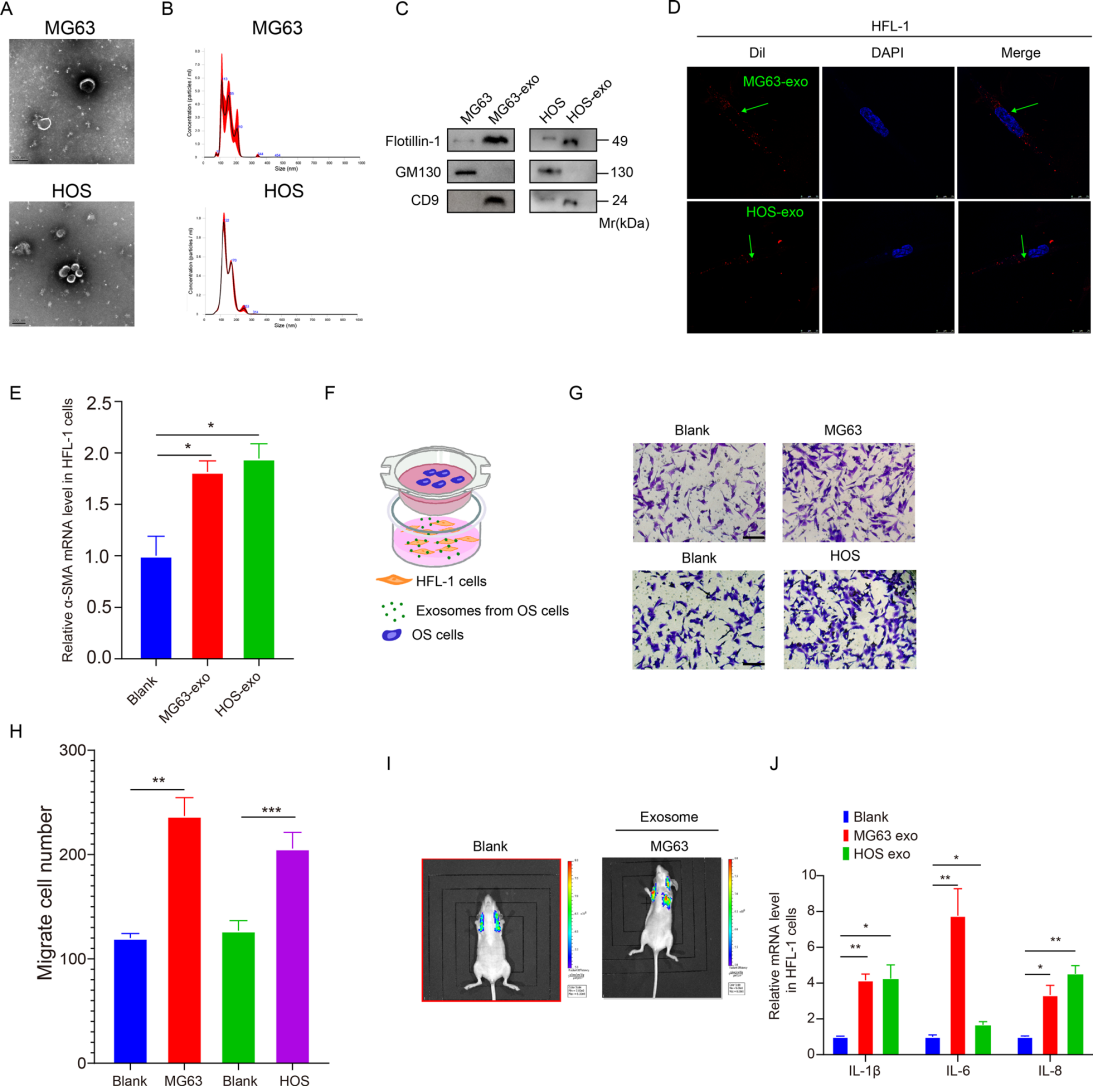
OS-derived exosomes induce lung fibroblasts activation and promote OS lung migration. **A**, **B**. Exosomes from MG63 and HOS cells were measured using electron microscopy and Nanosight particle tracking analysis. Scale bar: 100 nm. **C**. Western blot analysis was performed to examine the expression of marker proteins in exosomes derived from MG63 and HOS cells. **D**. Confocal imaging indicates that Dil-labeled exosomes (red) were delivered to DAPI-labeled HFL-1 cells (blue). The green arrow indicates the delivered exosomes and representative images. Scale bar: 25 μm. E. qRT-PCR detection of the expression of α-SMA in the exosomes-stimulated HFL-1 cells. F. Schematic diagram of cell co-culture *in-vitro* model. G and H. Representative images and quantitative analysis of transwell assay of OS cells migrated to HFL-1 cells when stimulated with OS-derived exosomes, respectively. I. Representative images of lung metastasis of mice stimulated with MG63-derived exosomes. J. qRT-PCR detection of the relative expression of IL-1β, IL-6, and IL-8 in HFL-1 cells stimulated with the two types of exosomes. (*p < 0.05; **p < 0.01; *** p < 0.0001)

Exosomes derived from two OS cell lines, HOS and MG63, were isolated and extracted to evaluate the involvement of tumor-derived exosomes in OS migration to the lung. Identifying these exosomes was accomplished through transmission electron microscopy and nanoparticle tracking analysis (NTA) techniques. Figure 1A, B show that the exosomes investigated in this study exhibit a circular morphology and possess a double-layered membrane. The size of those exosomes ranges from 100 to 147 nm, which is in line with the typical size range of exosomes. The results of the Western blot analysis showed an increase in the expression of proteins specific to exosomes (Flotillin-1 and CD9), while the expression of GM130 dramatically decreased, as shown in Fig. 1C.

HFL-1 cells (human lung fibroblast cells) were selected as normal fibroblasts (NFs) to study the impact of OS-derived exosomes in the activation of lung fibroblasts. OS-derived exosomes and HFL-1 cells were labeled with Dil (red) and DAPI (blue), respectively, to determine the transmission of exosomes. The confocal imaging findings indicated the existence of Dil spots in the recipient HFL-1 cells, indicating the transmission of labeled OS-derived exosomes to HFL-1 cells (Fig. 1D). qRT-PCR results showed the transcriptional upregulation of α-SMA in HFL-1 cells upon stimulation with exosomes secreted by two types of OS cells, indicating the activation of lung fibroblasts by OS-derived exosomes (Fig. 1E). Cell migration experiments indicated that exosome-simulated lung fibroblasts exhibited a higher propensity to induce OS migration (Fig. 1F–H). Significantly, intravenous injection of exosomes from MG63 cells significantly promoted OS cell-induced lung migration in vivo compared to the control group (Fig. 1I).

Moreover, fibroblasts cultured with exosomes derived from OS cells exhibited elevated expression of pro-inflammatory genes, including IL-1β, IL-6, and IL-8, which significantly influence the regulation of the inflammatory microenvironment and the advancement of cancer (Fig. 1J).

### OS-derived exosomal linc00881 promotes OS lung migration and induces lung fibroblasts activation

**Fig. 2:**
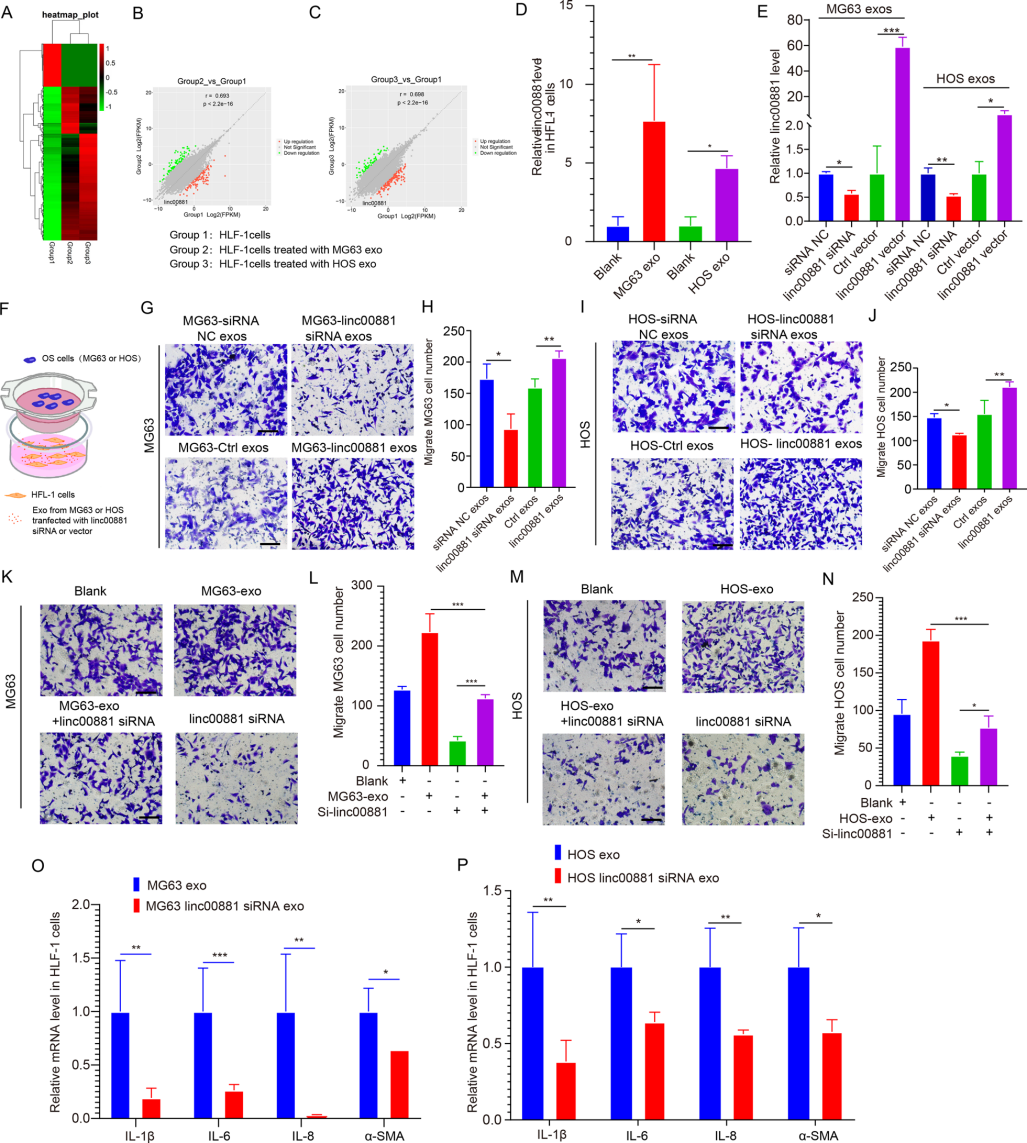
OS-derived exosomal linc00881 promotes OS lung migration and induces lung fibroblasts activation. **A**. RNA sequencing in a heatmap. **B**, **C**. Scatter plots of lncRNA expression in different groups. **D**. Relative expression of linc00881 in HFL-1 cells simulated by two OS exosomes. **E**. qRT-PCR detection of the expression linc00881 in exosomes with inhibited or overexpressed linc00881 in MG63 or HOS cells. **F**. Schematic diagram of the cell co-culture in vitro model. **G**–**J**. Representative images of MG63 (**G**) and HOS (**I**) cells migrated to HFL-1 cells with overexpressed or inhibited linc00881 in MG63 or HOS exosomes by transwell assay and the corresponding quantitative analysis (**H**, **J**). **K**–**N**. Representative images of MG63 (**K**) and HOS (**M**) cells migrated to HFL-1 cells with or without an inhibited expression of linc00881 in HFL-1 cells by transwell assay and the corresponding quantitative analysis (**L**, **N**). **O**, **P**. qRT-PCR detection of the relative expression of IL-1β, IL-6, IL-8, and α-SMA in HFL-1 cells with inhibited expression of linc00881 in MG63 or HOS exosomes. (*p < 0.05; **p < 0.01; *** p < 0.0001)

Next, we revealed the mechanism by which exosomes derived from OS activate lung fibroblasts. Exosomes contain a significant amount of lncRNA and are crucial in communicating between cells [24]. Thus, we hypothesize that tumor-derived exosomes lncRNAs mediate the activation of fibroblasts. To determine the specific lncRNA of OS exosomes, we stimulated lung fibroblasts with the exosomes derived from two distinct types of OS cells, followed by high-throughput sequencing, and then conducted high-throughput sequencing. Figure 2A displays the sequencing outcomes in the shape of a heat map. Furthermore, linc00881 shows a significant upregulation in lung fibroblasts upon stimulation with exosomes derived from two OS cells (Fig. 2B, C). Subsequently, qRT-PCR results indicated significant upregulation of linc00881 in lung fibroblasts when stimulated by exosomes originating from two OS cells (Fig. 2D). To demonstrate that linc00881 in exosomes promotes the migration of OS cells to lung fibroblasts, the expression of linc00881 was overexpressed or inhibited in the two OS cell lines. Subsequently, the aforementioned exosomes were isolated and extracted to stimulate lung fibroblasts. In MG63 and HOS cells, the upregulation or downregulation of linc00881 led to a corresponding change in linc00881 expression within the exosomes derived from OS cells (Fig. 2E). The migration capability of MG63 and HOS cells to lung fibroblasts was found to be decreased when the expression of linc00881 in exosomes was reduced, as shown by cell migration assays (Fig. 2F–J). Meanwhile, the increased expression linc00881 in exosomes resulted in the enhanced migration capability of MG63 and HOS cells to lung fibroblasts (Fig. 2F–J).

Subsequently, we examined the migratory potential of MG63 cells towards HFL-1 cells with the following experiment groups: (1) inhibition of linc00881 expression alone in HFL-1 cells, (2) OS-derived exosome-stimulated HFL-1 cells, (3) the combined treatment of (1) and (2), (4) the equal amount of PBS as the blank group. In the combined treatment group, the transwell assay demonstrated that the migratory capacity of MG63 and HOS cells towards HFL-1 cells was intermediate compared to the other two treatment groups. Furthermore, no notable distinction was noted compared to the control group (Fig. 2K–N).

Moreover, the inhibition of linc00881 in MG63 and HOS exosomes resulted in a reduced expression of pro-inflammatory genes, including IL-1β, IL-6, IL-8, and α-SMA, in both OS cell lines as compared to normal exosomes (Fig. 2O–P). These results indicated that linc00881 from OS cell-derived exosomes could activate OS lung migration and induce lung fibroblast.

### Linc00881 sponge miR-29c-3p in lung fibroblasts

**Fig. 3:**
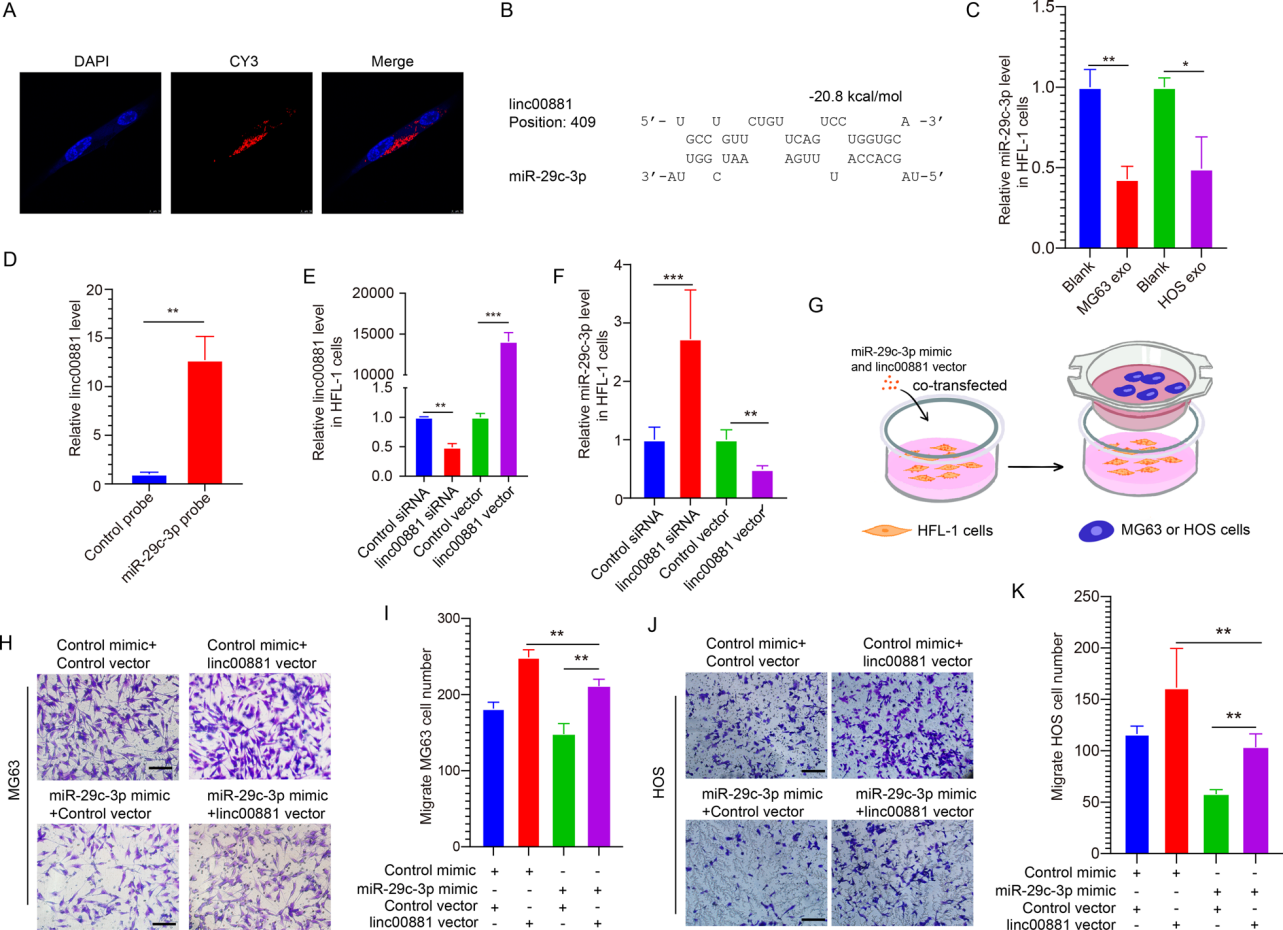
Linc00881 sponge miR-29c-3p in lung fibroblasts. **A**. Distribution of linc00881 in HFL-1 cells assessed by the FISH assay. **B**. Duplex structure of miR-29c-3p and linc00881. **C**. Relative expression of miR-29c-3p in HFL-1 cells simulated by two OS exosomes. **D**. qRT-PCR detection of relative expression of linc00881 using the control or miR-29c-3p probe to perform the pull-down assay. **E**. qRT-PCR detection of the expression of linc00881 in HFL-1 cells transfected with linc00881 siRNA or vector. **F**. qRT-PCR detection of the expression of miR-29c-3p in HFL-1 cells transfected as described in E. **G**–**K**. Transwell assay of MG63 or HOS cells migrated to the HFL-1 cells transfected with: a. control mimic + control vector; b. miR-29c-3p mimic + control vector; c. control mimic + linc00881 vector; d. miR-29c-3p mimic + linc00881 vector, representative images and quantitative analysis of MG63 and HOS cells are shown in H&I and J&K, respectively. (*p < 0.05; **p < 0.01; *** p < 0.0001)

The ceRNA mechanism is the most prevalent in regulating gene expression by tumor-derived exosomes [8, 25]. Therefore, the exosomes from OS may affect the lung migration of OS through the ceRNA mechanism. The FISH assay was performed on linc00881 in HFL-1 cells to validate the role of linc00881 as a miR-29c-3p sponge in HFL-1 cells. Figure 3A shows that linc00881 was localized in the cell cytoplasm, suggesting linc0081 may function as a ceRNA. We utilized the online software microcode (http://www.mircode.org/) and starbaseV2.0 (https://starbase.sysu.edu.cn/starbase2/) to predict the lncRNAs that can bind to miR-29c-3p. As presented in Fig. 3B, a potential interaction site was anticipated between linc00881 and miR-29c-3p. Subsequently, we found that linc00881 exhibits high expression levels in HFL-1 cells following stimulation with two OS exosomes (Fig. 3C). Subsequently, a biotin-avidin pull-down test verified that the linc00881 expression was elevated in the nucleotide complexes trapped by the miR-29c-3p probe when compared to the controls (Fig. 3D), indicating a potential interaction between linc00881 and miR-29c-3p. To demonstrate the correlation between linc00881 and miR-29c-3p in HFL-1 cells, we interfered/overexpressed linc00881 in lung fibroblasts. The expression level of miR-29c-3p significantly increased when linc00881 expression was interfered with, as demonstrated in Fig. 3E, F. The overexpression of linc00881 led to a reduction in the level of miR-29c-3p expression. The results above suggest that linc00881 forms a binding interaction with miR-29c-3p within HFL-1 cells. Subsequently, co-overexpression of linc00881 and miR-29c-3p was performed in HFL-1 cells to validate the role linc00881 in enhancing OS lung migration by sponging miR-29c-3p in HFL-1 cells. The transwell assay showed that the increased expression of miR-29c-3p could reverse the impact of linc00881 overexpression on the migration of OS cells in the lungs (Fig. 3G–K). The findings indicated that linc00881 affects OS lung migration by sponging miR-29c-3p in HFL-1 cells.

### miR-29c-3p regulates the expression of MMP2 in lung fibroblasts cells

**Fig. 4:**
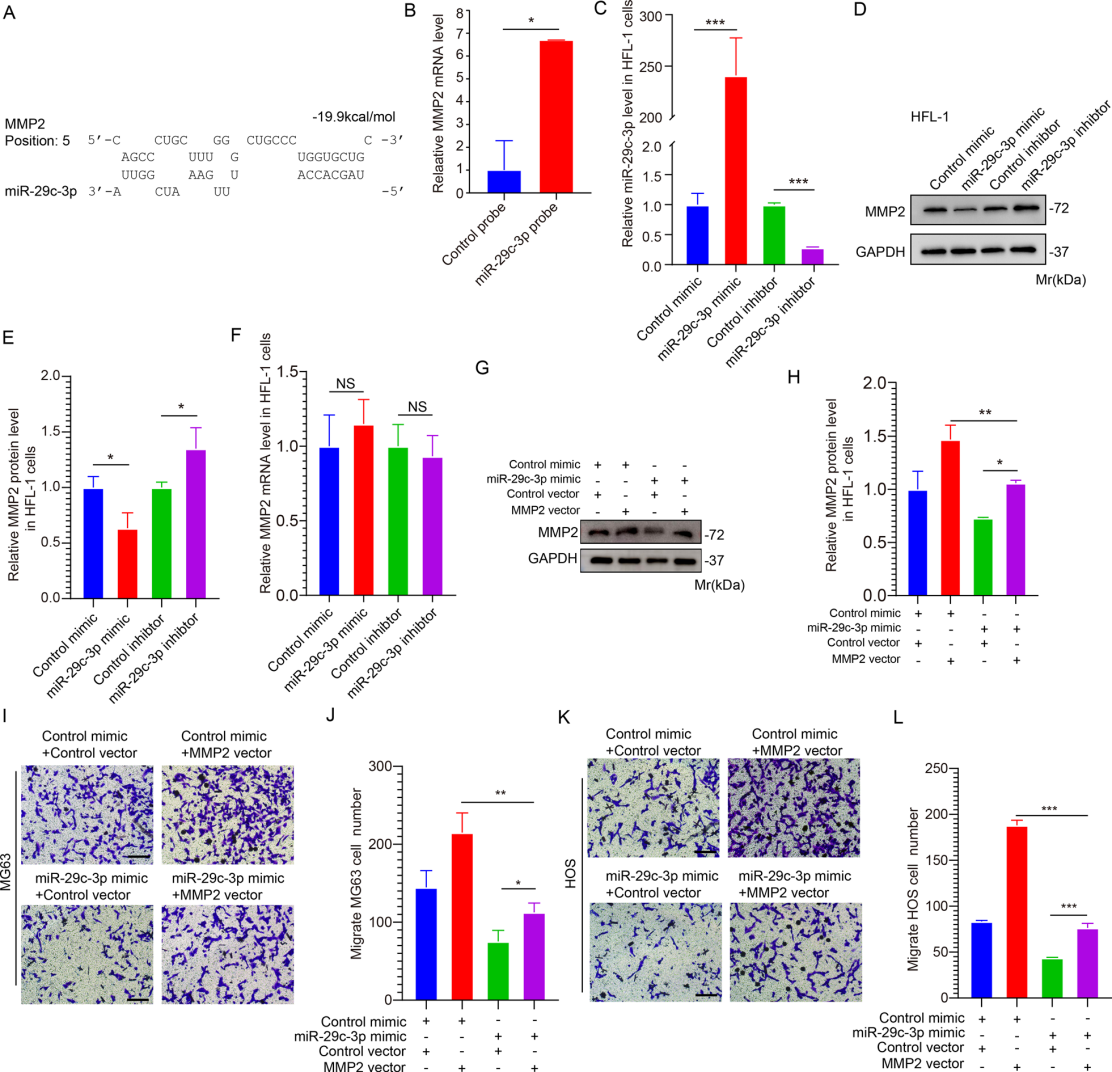
miR-29c-3p directly regulates the expression of MMP2 in lung fibroblasts cells. **A**. Duplex structure of MMP2 3′-UTR (Untranslated Region) and miR-29c-3p. **B**. The relative expression of MMP2 was detected at the mRNA level by qRT-PCR using a control or miR-125a-5p probe to perform the pull-down assay. **C**. The miR-29c-3p expression was detected via qRT-PCR after the control/miR-29c-3p mimic or control/miR-29c-3p inhibitor was transfected into HFL-1 cells. **D**–**F**. Representative images (**D**) and quantitative analysis (**E**, **F**) of Western blotting analysis and qRT-PCR of MMP2 expression in HFL-1 cells after transfected as described in **C**. **G**, **H**. Representative images (**G**) and quantitative analysis (**H**) of Western blotting analysis of MMP2 expression in HFL-1 cells transfected as follows: a. control mimic and control vector; b. control mimic and the miR-29c-3p-overexpressed plasmid; c. miR-29c-3p mimic and control vector; d. miR-29c-3p mimic and MMP2 vector. **I**–**L**. Representative images (**I** MG63, **K** HOS) and quantitative analysis (**J**, **L**) of transwell assay of MG63 and HOS cells migrated to HFL-1 cells after transfected as described in **G**. (*p < 0.05; **p < 0.01; *** p < 0.0001)

To identify the downstream regulatory proteins of miR-29c-3p, three types of bioinformatics software were utilized to predict the target gene of miR-29c-3p [26, 27]. Using various software tools, miR-29c-3p was found to target matrix metalloproteinase 2 (MMP2), a significant member of the matrix metalloproteinase family [28]. Invasive tumor cells are known to have a strong ability to degrade extracellular matrix through the activation of MMP2 [29]. MMP2 promotes metastasis in several types of cancer, including gastric cancer [30], ovarian cancer [31], breast cancer [32], and OS [33]. The predicted docking site of MMP2 to miR-29c-3p is shown in Fig. 4A. The calculated binding energy between miR-29c-3p and MMP2 was − 19.9 kJ/mol, within the range of binding energy between miRNAs and target genes. A biotin pull-down test was conducted to validate the binding, which revealed that the miR-29c-3p probe captured nucleotide complexes with higher expression of MMP2 mRNA than the control group (Fig. 4B). The findings indicated that miR-29c-3p directly targets the gene MMP2.

To demonstrate that miR-29c-3p can regulate the expression of MMP2 in HFL-1 cells, the overexpression or inhibition of miR-29c-3p was performed in HFL-1 cells (Fig. 4C). As shown in Fig. 4D, E, the overexpression and inhibition of miR-29c-3p can decrease or increase the expression of MMP2, respectively. However, miR-29c-3p regulates the MMP2 expression in HFL-1 cells at the post-transcriptional level. This is evident from the fact that the mRNA level of MMP2 remained unchanged despite overexpression or suppression of miR-29c-3p (Fig. 4F). The transwell assay demonstrated that miR-29c-3p could suppress the movement of OS cells toward HFL-1 cells (Additional file 1: Fig S1A–E). Additional file 2: Fig S2A–F suggests that inhibiting/overexpressing MMP2 expression in HFL-1 cells leads to a decrease/increase in the migration capability of MG63 and HOS cells. Subsequently, our findings demonstrated that the increased expression of miR-29c-3p suppressed the levels of α-SMA, IL-1β, IL-8, and IL-6 (Additional file 1: Fig. S1F). Furthermore, the overexpression of MMP2 led to an enhanced migration capability of both MG63 and HOS cells. Moreover, the suppression of MMP2 in HFL-1 cells reduced the levels of α-SMA, IL-1β, IL-8, and IL-6 by qRT-PCR (Additional file 2: Fig. S2I). The overexpression of MMP2 led to elevated expression of α-SMA, IL-1β, IL-8, and IL-6 (Additional file 2: Fig. S2J).

To further verify the regulation of MMP2 expression by miR-29c-3p, miR-29c-3p, and MMP2 were co-overexpressed in HFL-1 cells. Western blotting results displayed in Fig. 4G, H indicated that the expression of MMP2 did not increase in the co-overexpression model. These results suggested that the overexpression of miR-29c-3p could rescue the effect caused by the overexpression of MMP2 alone. Finally, the transwell assay rescue experiment demonstrated that the overexpression of miR-29c-3p could rescue the migratory effect caused by the overexpression of MMP2 alone in the two OS cell lines (Fig. 4I–L). The above results indicated that miR-29c-3p regulates the post-transcriptional expression of MMP2 and inhibits the migration of OS cells toward HFL-1 cells.

### Exosomal Linc00881 regulates the expression of MMP2 in lung fibroblasts by sponging miR-29c-3p

**Fig. 5:**
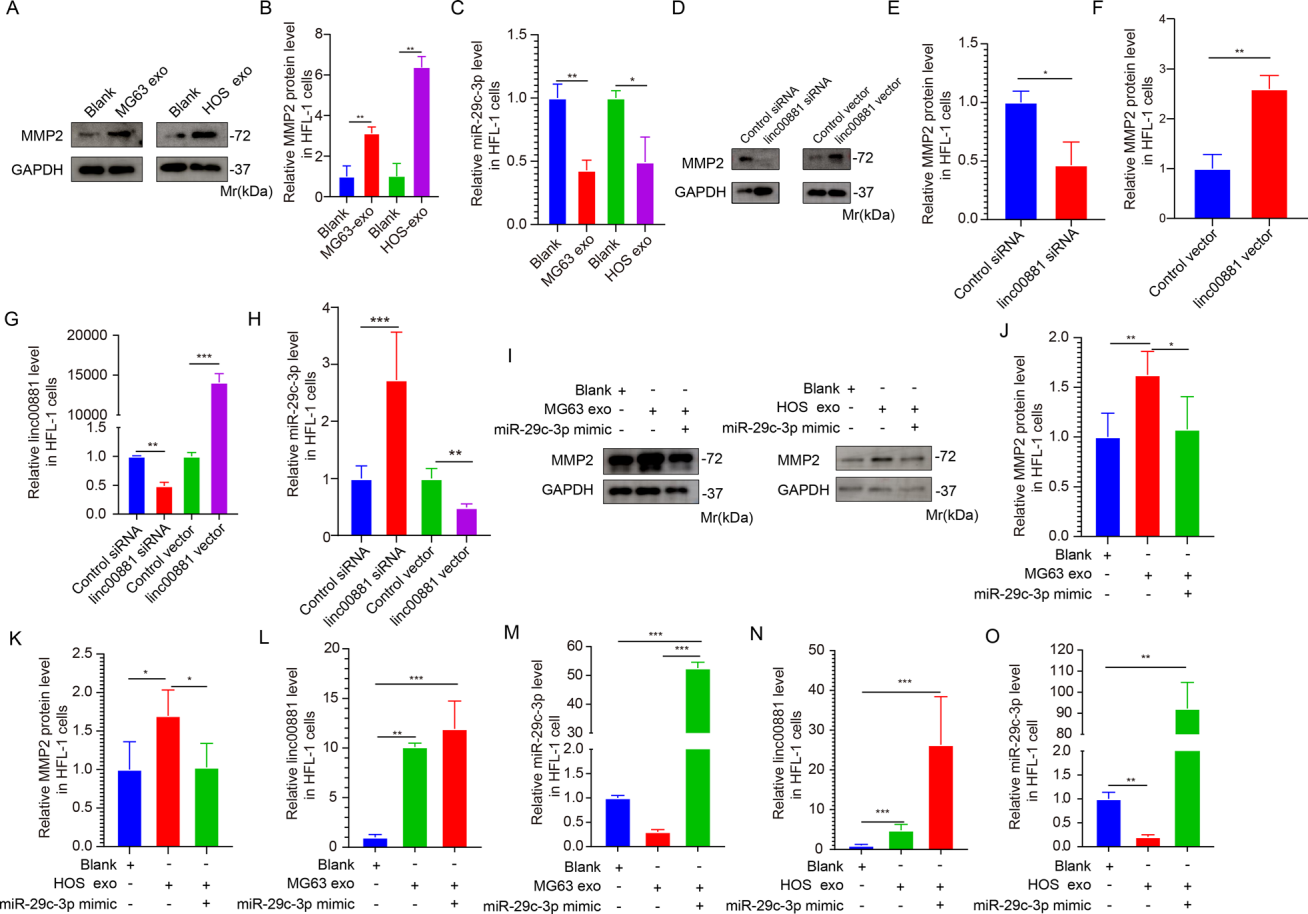
Exosomal Linc00881 regulates the expression of MMP2 in lung fibroblasts by sponging miR-29c-3p. **A**, **B**. Representative images (**A**) of Western blotting analysis of MMP2 expression in HFL-1 cells treated with blank, MG63, or HOS exosomes and the corresponding quantitative analysis (**B**). **C**. Relative miR-29c-3p concentration was detected in HFL-1 cells simulated by the two types of OS-derived exosomes. **D**-**F**. Representative images (**D**) of Western blotting analysis of MMP2 expression in HFL-1 cells transfected with linc00881 siRNA or vector and the corresponding quantitative analysis (**E**, **F**). **G**. qRT-PCR detection of the expression of linc00881 in HFL-1 cells transfected with linc00881 siRNA or vector. **H**. qRT-PCR detection of the expression of miR-29c-3p in HFL-1 cells transfected as described in **B**. **I**–**K**. Representative images (**I**) of Western blotting analysis of MMP2 expression in HFL-1 cells transfected with miR-29c-3p mimic and stimulated with MG63 or HOS cells-derived exosomes, and the corresponding quantitative analysis (**J**, **K**). **L**–**O**. qRT-PCR detection of the relative expression of linc00881 or miR-29c-3p in HFL-1 cells treated in **I**. (*p < 0.05; **p < 0.01; *** p < 0.0001)

Figure 5A–C demonstrate a significantly higher expression of MMP2 in HFL-1 cells stimulated by OS exosomes compared to the control group (Fig. 5A–C). To demonstrate that linc00881 regulates MMP2 expression by sponging miR-29c-3p in lung fibroblasts, we inhibited or overexpressed the expression of linc00881 in HFL-1 cells. The results indicate that inhibition of linc00881 expression led to a downregulation of MMP2 expression compared to the control group (Fig. 5D–F). When linc00881 was overexpressed, the expression of MMP2 increased compared to the control group (Fig. 5D–F).

Subsequently, qRT-PCR was employed to measure the levels of linc00881 and miR-29c-3p in HFL-1 cells. The findings indicated that suppression of linc00881 led to a reduction in its expression and a rise in miR-29c-3p expression (Fig. 5G, H). In contrast, overexpression of linc00881 elevation in linc00881 expression and a reduction in miR-29c-3p expression (Fig. 5G, H). The findings indicated that linc00881 could control the MMP2 expression by acting as a sponge for miR-29c-3p in HFL-1 cells. Furthermore, the expression of miR-29c-3p was downregulated in the exosome-treated group (Fig. 5C). These results suggested that OS-exosomal linc00881 enhances the expression of MMP2 by regulating miR-29c-3p expression.

To verify that OS-exosomal linc00881 regulates MMP2 expression by sponging miR-29c-3p, miR-29c-3p was overexpressed in HFL-1 cells and exosomes from MG63 and HOS cells were employed to induce stimulation in HFL-1 cells. Western blotting and qRT-PCR were utilized to detect the presence of MMP2 protein and miR-29c-3p expression, respectively. The results revealed that overexpression of miR-29c-3p could rescue the effect of OS-derived exosomes on the expression of MMP2 in HFL-1 cells (Fig. 5I, K). Exposing HFL-1 cells to exosomes derived from OS led to enhanced linc00881 expression (Fig. 5L, N). Moreover, the upregulation of miR-29c-3p in HFL-1 cells can mitigate the influence of exosomes derived from oxidative stress on the miR-29c-3p expression (Fig. 5M, O). These results suggested that OS exosomal linc00881 upregulates the expression of MMP2 by adsorbing miR-29c-3p in HFL-1 cells and activating lung migration.

### Linc00881 activates lung fibroblasts through the NF-κB axis

**Fig. 6:**
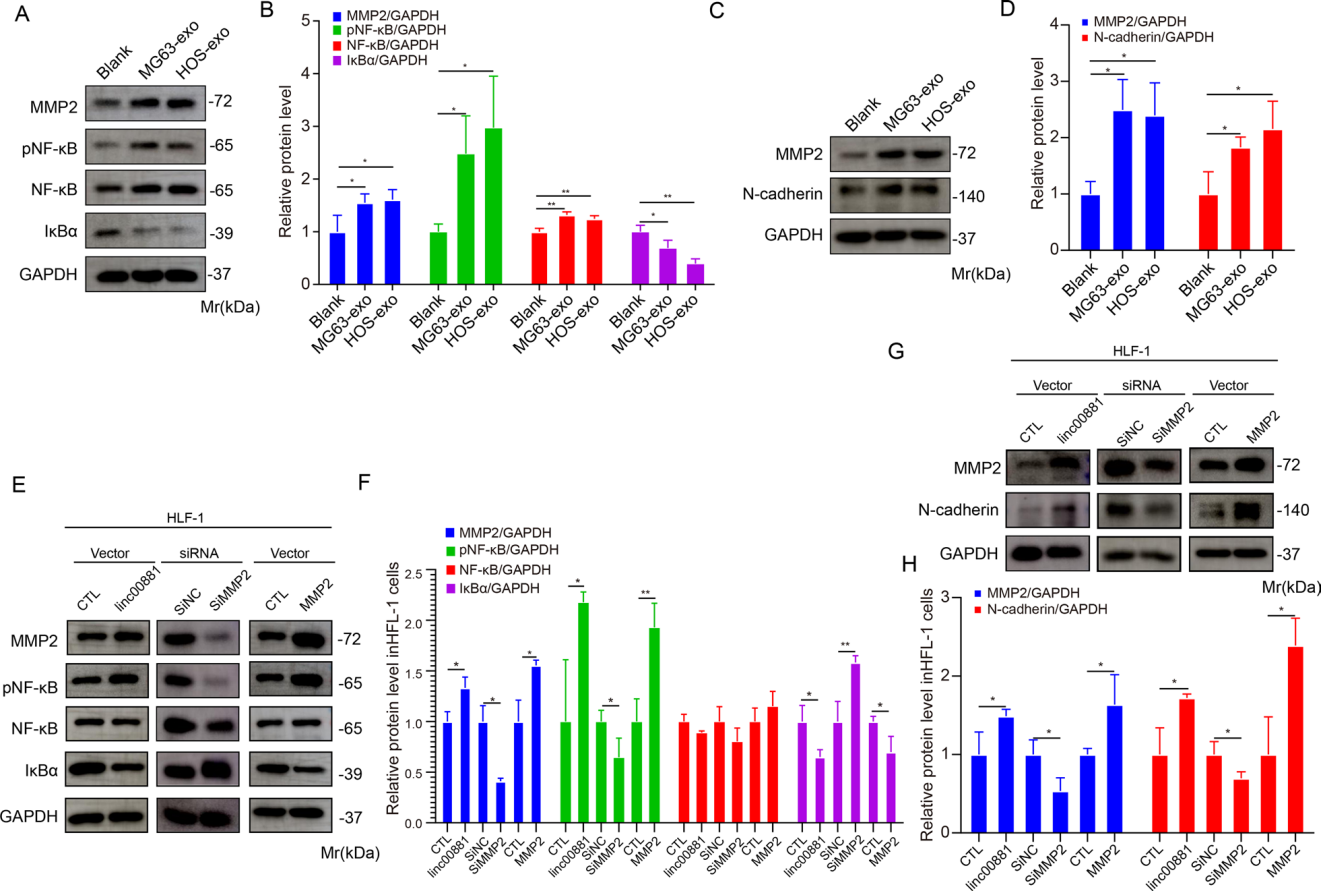
Linc00881 activates lung fibroblasts through the NF-κB axis. **A**, **B**. Western blotting analysis of the proteins of NF-κB axis in HFL-1 cells treated with exosomes from MG63 and HOS cells. **C**, **D**. Western blotting analysis of the expression of MMP2 and N-cadherin in HFL-1 cells treated with MG63 or HOS cell-derived exosomes. **E**, **F**. Western blotting analysis of the proteins of NF-κB axis in HFL-1 cells treated with: a. control vector, b. linc00881 vector, c. control siRNA, d. MMP2 siRNA, e. control MMP2, and f. MMP2 vector. **G**, **H**. Western blotting analysis of the expression of MMP2 and N-cadherin in HFL-1 cells in different treatments as described in **E**. (*p < 0.05; **p < 0.01; *** p < 0.0001)

These results indicate that linc00881 promoted the pro-inflammatory factors IL-1β, IL-6, and IL-8 in HFL-1 cells. Previous studies have demonstrated that these pro-inflammatory factors are targets of NF-κB (nuclear factor-κB) [34]. Therefore, we investigated the NF-κB signaling pathway in this experimental group. The exosomes obtained from the two OS cell lines enhanced the phosphorylated NF-κB expression in HFL-1 cells and reduced the expression of IκBα (inhibitor of NF-κB) (Fig. 6A, B) as compared to the blank group, suggesting OS-derived exosomes played a role in activating the NF-κB signaling pathway.

To confirm the activation of the NF-κB signaling pathway by exosomal linc00881 in OS, linc00881 was upregulated in OS cells, and exosomes were extracted. As shown in Fig. 6E, F, inhibition of linc00881 in exosomes led to an increase in phosphorylated NF-κB expression and a decrease in IκBα expression compared to the control. The result is consistent with the impact of MMP2 overexpression on the NF-κB signaling pathway, suggesting that OS exosomal linc00881 activates the NF-κB signaling pathway in HFL-1 cells.

N-cadherin, a downstream protein of MMP2, plays a vital part in promoting the process of Epithelial-mesenchymal transition (EMT). Subsequently, western blotting was employed to measure the protein expression of MMP2 and N-cadherin in HFL-1 cells. The results demonstrated that treatment of HFL-1 cells with OS-derived exosomes significantly increased the levels of N-cadherin and MMP2 (Fig. 6C, D). When MMP2 was upregulated or downregulated in HFL-1 cells, the phosphorylated NF-κB expression and IκBα expression have been changed compared to the control (Fig. 6G, H). These results indicated that OS exosomal linc00881 increased the expression of MMP2, leading to an increase in N-cadherin protein levels and activating the NF-κB signaling pathway in HFL-1 cells.

### Activated fibroblasts by exosomal linc00881 accelerate OS progression

**Fig. 7:**
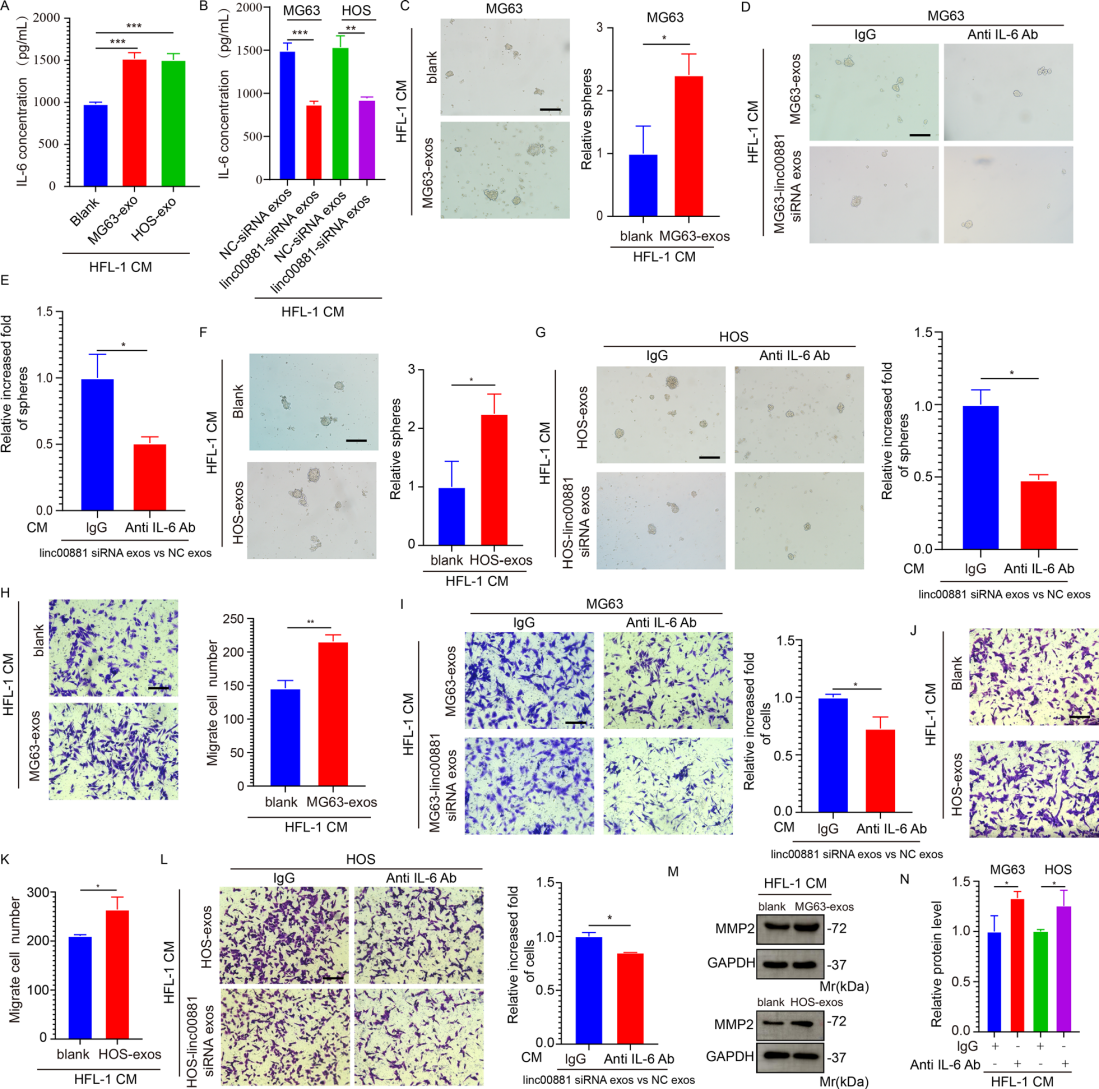
Activated fibroblasts by exosomal linc00881 accelerate OS progression. **A**. IL-6 secretion from HFL-1 treated by exosomes from two OS cells was detected via ELISA assay. **B**. IL-6 secretion from HFL-1 treated by exosomes from two OS cells or exosomes from linc00881 interrupted two OS cells and was detected via ELISA assay. **C**. Spheroid formation ability of MG63 treated with indicated CM. Representative Images and quantitative analysis were shown.100 × . **D**–**E**. Spheroid formation ability of MG63 treated with CM containing: a. IL-6 neutralizing antibody, b. IgG control antibody, c. linc00881 siRNA exo, d. IL-6 neutralizing antibody plus linc00881 siRNA exo. Representative Images and quantitative analysis were shown.100 × . **F**. Spheroid formation ability of HOS treated with indicated CM. Representative Images and quantitative analysis were shown.100 × . **G**. Spheroid formation ability of MG63 treated with CM as indicated in **D**. Representative Images and quantitative analysis were shown.100 × . **H**. Migration assay of MG63 treated with indicated CM. Representative Images and quantitative analysis were shown.100 × . **I**. Migration assay of MG63 treated with indicated CM indicate in **D**. Representative Images and quantitative analysis were shown.100 × . **J**, **K**. Migration assay of HOS treated with indicated CM. Representative Images and quantitative analysis were shown.100 × . **L**. Migration assay of HOS treated with CM indicated in **D**. Representative Images and quantitative analysis were shown.100 × . **M**, **N**. Western blotting analysis of the expression of MMP2 in MG63 and HOS cells treated with indicated CM. (*p < 0.05; **p < 0.01; *** p < 0.0001)

The release of various pro-inflammatory cytokines and chemokines by CAFs is widely recognized as crucial for tumor progression [35]. To investigate whether exosomal linc00881-educated fibroblasts play a role in promoting tumor characteristics, a series of in vitro experiments were performed. Firstly, it was observed that the IL-6 levels in HFL-1 cells rose following the exosome stimulation from MG63 and HOS cells (Fig. 7A). We observed that interference with linc00881 exosomes decreased IL-6 secretion in HFL-1 cells compared to the normal exosomes group (Fig. 7B). Subsequently, MG63 and HOS cells were cultured using CM collected from HFL-1 pretreated with PBS or exosomes from two OS cells. After treatment, tumor cells exhibited increased expression of spheroid formation ability and motility (Fig. 7C, F, H, J, K). Significantly, the enhanced spheroid formation and motility of OS cells, resulting from incubation with conditioned medium (CM) collected from fibroblasts pretreated with OS exosome, were partially mitigated by blocking IL-6 using neutralizing antibodies (Fig. 7D, E, G, I, L).

Subsequently, tumor cells were cultured using CM collected from HFL-1 pretreated with exosomes and exosomes interfering with linc00881. After treatment, tumor cells in the exosomes pre-treated group exhibited a reduced ability to form spheroids and to migrate, compared to the group pre-treated with exosomes interfering with linc00881 (Fig. 7D, E, G, I, L). Significantly, the enhanced spheroid formation and motility of OS cells, following exposure to CM obtained from fibroblasts pre-treated with OS exosome, were partially reversed by the inhibition of IL-6 using neutralizing antibodies (Fig. 7D, E, G, I, L). Western blot analysis showed that the expression of MMP2 in tumor cells was significantly increased following pre-treatment with exosomes from two OS cells (Fig. 7M, N). These results indicate that fibroblasts cultured with linc00881 derived from OS exosomes may enhance the proliferation and migration of cancer stem cells.

## Discussion

Intercellular communication coordinates the dynamic system of the tumor microenvironment (TME), significantly contributing to tumor progression [36]. Exosomes derived from cancer significantly impact the control of TME [37]. Therefore, it is crucial to investigate the interaction between tumors and the extracellular matrix facilitated by exosomes. This study discovered that OS-derived exosomes promoted OS lung migration and converted lung fibroblasts into CAFs. Subsequently, we performed high-throughput sequencing on lung fibroblasts stimulated by exosomes from two OS cells. The results indicated that exosomal linc00881, a novel lncRNA, may be involved in the process of lung migration in OS. Our study verified that linc00881 transfers directly from OS cells to fibroblasts in the pre-metastatic niche of the lung through exosomes and activates the NF-κB signaling pathway by upregulating the MMP2 expression to convert fibroblasts into CAFs. Additionally, CAFs aggravate tumor development by secreting IL-1β, IL-6, and IL-8. The reciprocal communication between cancer cells and fibroblasts enhances our understanding of the molecular mechanisms underlying lung metastasis in cancer and provides insights into the specific mechanisms by which OS cells migrate to the lungs.

Limited research has been conducted on exosomal lncRNAs in OS, with the primary emphasis on the progression of OS. For example, the exosomal lncRNA ANCR of OS has been shown to enhance drug resistance [38]. OS exosomal RNA ELFN1-AS1 accelerates OS tumorigenesis [25]. OS exosomal linc00852 promotes OS progression [39]. Our results indicate that the exosomal linc00881 derived from OS cells transforms lung fibroblasts into CAFs within the pre-metastatic niche of the lungs. Subsequently, we confirmed the relationship between linc00881 and miR-29c-3p, which was initially predicted by bioinformatics and later verified through an RNA pull-down assay. Subsequently, we observed an increase in the expression of linc00881 and a decrease in the expression of miR-29c-3p in HFL-1 cells. Stimulating HFL-1 cells with exosomes derived from OS cells resulted in the direct transfer of linc00881 in its exosomal form, which occurred before lung migration, consequently inducing fibroblast transformation into CAFs. Subsequently, CAF can alter the tumor microenvironment by secreting IL-1β, IL-6, and IL-8, thereby enhancing the migration of OS cells. Finally, our findings indicated that linc00881 promoted the MMP2 expression by adsorbing miR-29c-3p in HFL-1 cells, activating the NF-κB signaling pathway and further altering the tumor microenvironment.

MMP2, a cancer-causing gene, significantly impacts the movement of cells, tumor microenvironment, and angiogenesis [40]. Increased expression of MMP2 results in extracellular matrix degradation and promotes the release of inflammatory factors [41, 42]. Our data indicate that MMP2 in lung fibroblasts is regulated by linc00881 of exosomes derived from OS, which promotes the release of cytokines, thus activating the NF-KB signaling pathway and establishing the microenvironment before tumor metastasis. The mechanism of CAFs in cancer metastasis is very complex, and the role of CAFs in lung metastasis of osteosarcoma requires further investigation.

In conclusion, our findings showed that the OS-derived exosomal linc00881 could induce the transformation of lung fibroblasts into CAFs with increased secretion of IL-1β, IL-6, and IL-8. Subsequently, our results have shown that OS-derived exosomal linc00881 promotes OS lung migration by upregulating the expression of MMP2. The adsorption of miR-29c-3p and activation of the NF-κB signaling pathway mediates this effect. Our study revealed a novel molecular mechanism by which the interaction between OS cells and fibroblasts promotes lung migration, thus facilitating the advancement of early diagnostic methods and effective treatment strategies for OS.

### Supplementary Information

**Additional file 1: Figure S1.** miR-29c-3p promotes OS lung migration and inhibits the activation of lung fibroblasts. A. Schematic diagram of the cell co-culture in vitro model. B-E. Transwell assay of MG63 or HOS cells migrated to HFL-1 cells transfected with miR-29c-3p mimic or inhibitor. Representative images and quantitative analysis of MG63 and HOS cells are shown in B&C and D&E, respectively. F. Relative expression of IL-1β, IL-6, IL-8, and α-SMA in miR-29c-3p overexpressed HFL-1 cells as detected by qRT-PCR. (*p < 0.05, **p < 0.01).

**Additional file 2: Figure S2.** MMP2 induces lung fibroblasts activation and promotes OS lung migration. A and B. Representative images and quantitative analysis of Western blotting analysis of MMP2 expression in HFL-1 cells transfected with control and MMP2 siRNA, respectively. C and D. Representative images and quantitative analysis of Western blotting analysis of MMP2 expression in HFL-1 cells transfected with control vector and MMP2 vector, respectively. E-H. Representative images and quantitative analysis of transwell assay of MG63 (E and F) or HOS (G and H) cells migrated to HFL-1 cells transfected with control siRNA, MMP2 siRNA, control vector, or MMP2 vector. I and J. qRT-PCR detection of relative expression of IL-1β, IL-6, IL-8, and α-SMA in HFL-1 cells with MMP2 be interfered or overexpressed. (*p < 0.05; **p < 0.01; *** p < 0.0001),

## Data Availability

All the data are presented in the manuscript. If anyone needs to obtain the original data, they can contact Associate Professor Yan Liang privately by email at lyan@wnmc.edu.cn.

## Abbreviations

OS: Osteosarcoma
CAFs: Cancer-associated fibroblasts
MMP2: Matrix metalloproteinases 2
qRT-PCR: Quantitative reverse transcription PCR
NF: Normal fibroblasts
