# Supplementary figures and images for: Tumor-derived exosomal linc00881 induces lung fibroblast activation and promotes osteosarcoma lung migration
Source: Cancer Cell Int. 2023 Nov 21;23:287. doi: 10.1186/s12935-023-03121-3 (PMC10664679; doi:10.1186/s12935-023-03121-3)

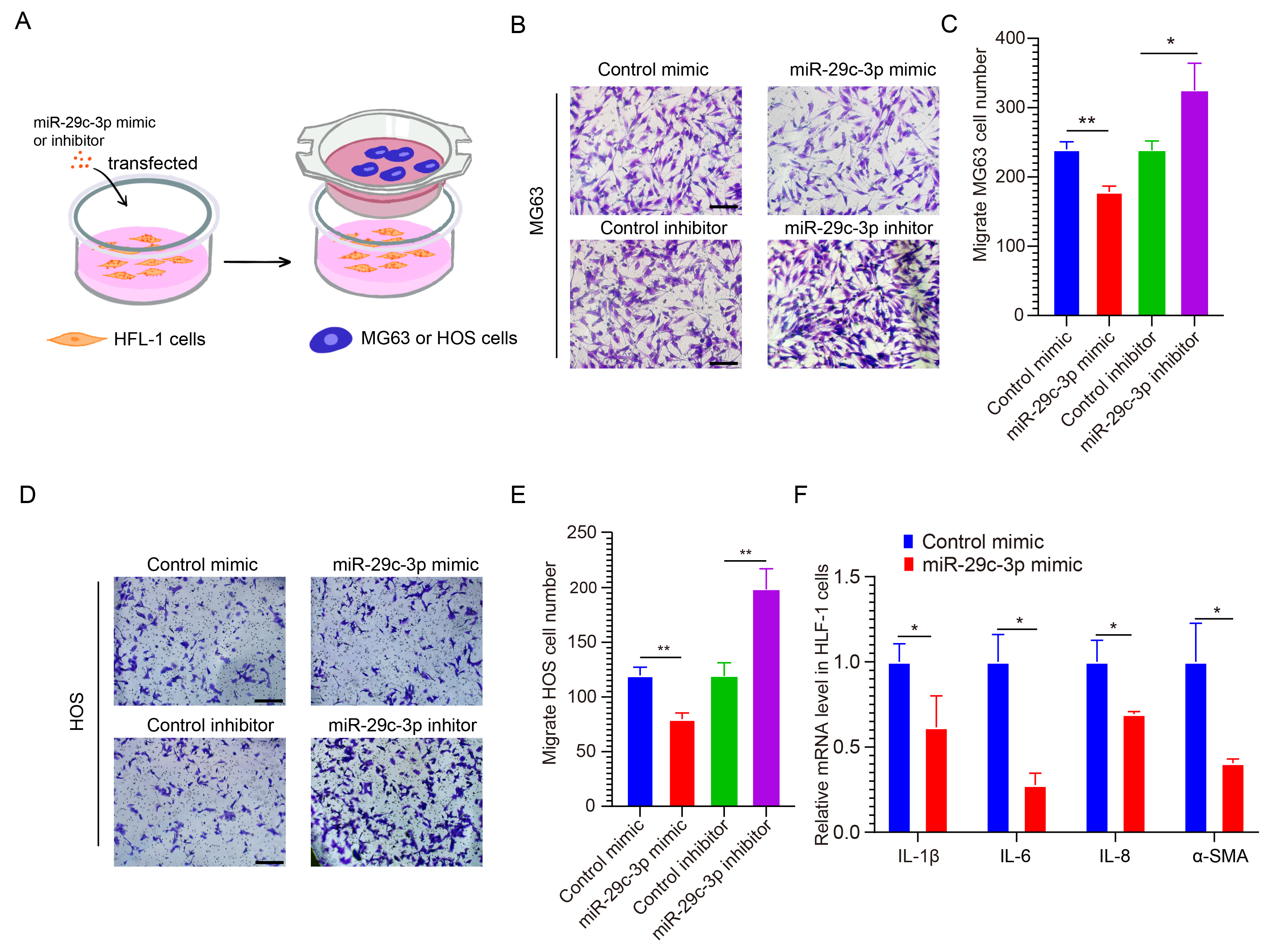

Supplement: Supplementary file 1 — Additional file 1: Figure S1. miR-29c-3p promotes OS lung migration and inhibits the activation of lung fibroblasts. A. Schematic diagram of the cell co-culture in vitro model. B-E. Transwell assay of MG63 or HOS cells migrated to HFL-1 cells transfected with miR-29c-3p mimic or inhibitor. Representative images and quantitative analysis of MG63 and HOS cells are shown in B&C and D&E, respectively. F. Relative expression of IL-1β, IL-6, IL-8, and α-SMA in miR-29c-3p overexpressed HFL-1 cells as detected by qRT-PCR. (*p < 0.05, **p < 0.01). [file 12935_2023_3121_MOESM1_ESM.tif]

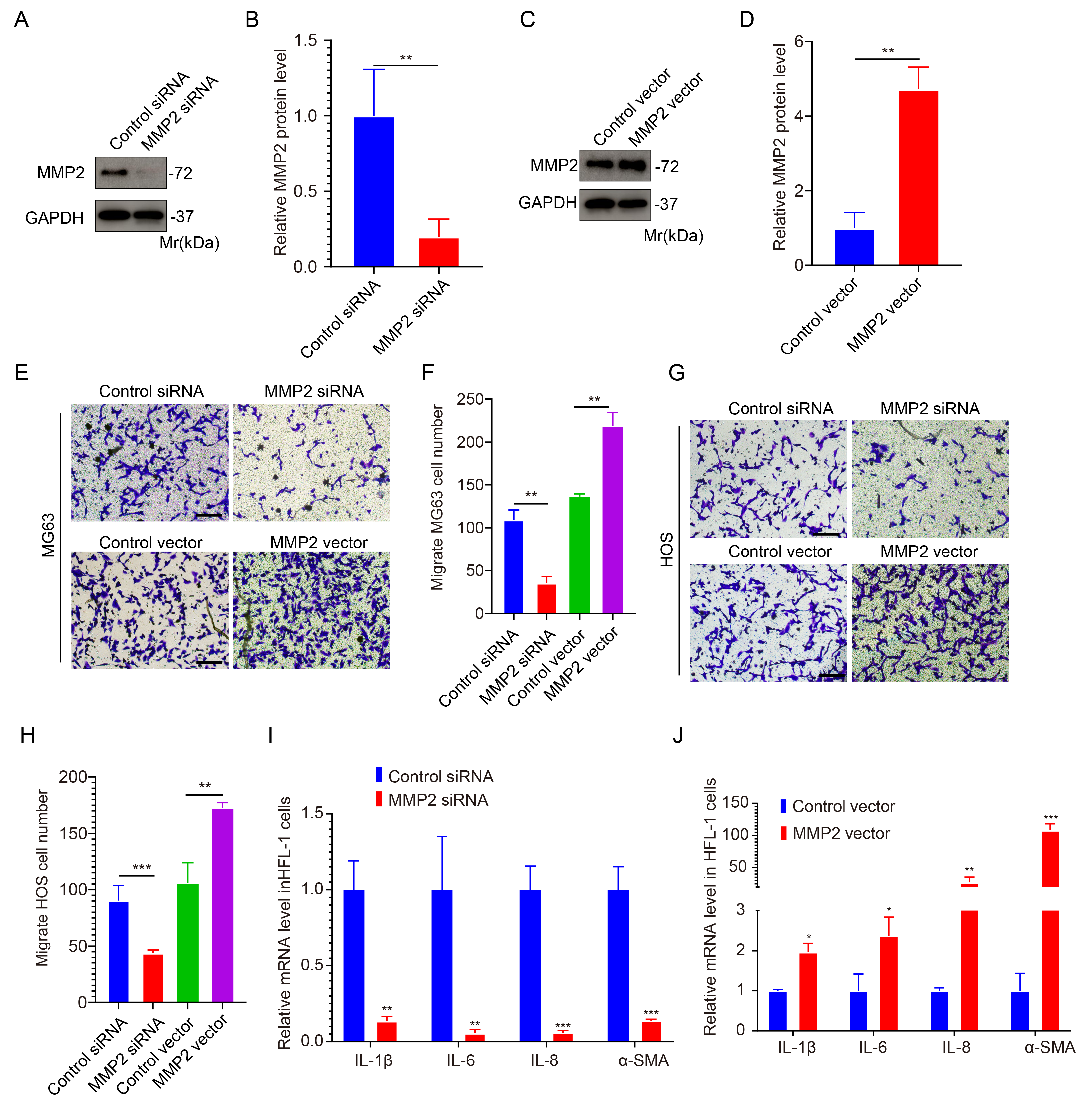

Supplement: Supplementary file 2 — Additional file 2: Figure S2. MMP2 induces lung fibroblasts activation and promotes OS lung migration. A and B. Representative images and quantitative analysis of Western blotting analysis of MMP2 expression in HFL-1 cells transfected with control and MMP2 siRNA, respectively. C and D. Representative images and quantitative analysis of Western blotting analysis of MMP2 expression in HFL-1 cells transfected with control vector and MMP2 vector, respectively. E-H. Representative images and quantitative analysis of transwell assay of MG63 (E and F) or HOS (G and H) cells migrated to HFL-1 cells transfected with control siRNA, MMP2 siRNA, control vector, or MMP2 vector. I and J. qRT-PCR detection of relative expression of IL-1β, IL-6, IL-8, and α-SMA in HFL-1 cells with MMP2 be interfered or overexpressed. (*p < 0.05; **p < 0.01; *** p < 0.0001), [file 12935_2023_3121_MOESM2_ESM.tif]
